# Prevalence of knowledge on maternal physical activity among pregnant women: a protocol for a systematic review

**DOI:** 10.1186/s44167-022-00006-0

**Published:** 2022-11-02

**Authors:** Madhawa Perera, Kumara Dissanayake, Lalith Senarathna

**Affiliations:** 1 Department of Gynaecology and Obstetrics, Faculty of Medicine and Allied Sciences, Rajarata University of Sri Lanka, Anuradhapura, Sri Lanka; 2 Department of Health Promotion, Faculty of Applied Sciences, Rajarata University of Sri Lanka, Anuradhapura, Sri Lanka

**Keywords:** Maternal physical activity, Knowledge, Pregnant women, Pregnancy

## Abstract

**Background:**

Maternal physical activity is beneficial to pregnant women, fetus and newborns. Evidence suggests that the level of physical activity in this group is not up to the expectation around the world. Lack of knowledge on the benefits of physical activity during pregnancy and not being aware of the recommendations are major reasons for this situation. Although individual studies have reported various levels of knowledge on maternal physical activity in different populations, no studies have systematically reviewed the literature to provide global evidence on the topic that is useful in initiating multinational approaches to improve maternal physical activity. The proposed study aims to systematically review prevalence of knowledge on maternal physical activity among pregnant women in different regions in the world.

**Methods:**

The proposed systematic review is designed according to the guidelines for conducting systematic reviews of prevalence and will be reported following the recommendations in the PRISMA statement. Quantitative cross-sectional, descriptive and observational studies published from year 2000 to 2022 will be included in the review. PubMed, Scopus, CINAHL, Embase, SPORTDiscus and Web of Science Core Collections will be searched using keywords relevant to physical activity, pregnant women and knowledge. Grey literature on the topic will be located through searching grey information sources, hand searching of reference lists and communicating with experts in the field. Screening of search results, selection and quality assessment of studies and data extraction will be independently performed by two reviewers. Assistance of a third reviewer will be sought to resolve any disagreement during the selection and quality assessment steps. After appraising the quality and consistency of selected studies, a premade data collection form will be used for data extraction. Narrative synthesis approach will be used in this review to analyze the evidence in primary studies.

**Discussion:**

The proposed review will summarize evidence on the level of knowledge on maternal physical activity among pregnant women in different populations and delineate interregional discrepancies. The study will locate high priority regions with poor knowledge and identify elements of knowledge that needs attention.

**Supplementary Information:**

The online version contains supplementary material available at 10.1186/s44167-022-00006-0.

## Background

Physical activity and exercise during pregnancy have been considered as undesirable practices for long time owing to lack of empirical evidence on the safety, influence of diverse socio-cultural believes and attitudes that promote sedentary lifestyles among pregnant women [1, 2]. However, as the scientific evidence continued to show that physical activity and exercise during pregnancy could prevent pre-eclampsia [3], gestational diabetes mellitus [4], excessive gestational weight gain [5], depressive disorders [6] and reduce the risk of instrumental [7] or cesarean delivery [8], the traditional requirement of taking a “pregnant pause” in terms of participation in exercise was empirically attenuated [9].

Contemporary evidence revealed that maternal physical activity is not only beneficial to mothers, but also to the fetus and the newborn. Antenatal exercise reduces the risk of macrosomia [10, 11], increase the rate of live births [12] and improve children’s neurodevelopment [13]. Maternal physical activity also has the ability of combating the vicious cycle of noncommunicable diseases by influencing the fetal programing in the womb [14]. It is now clear that exercise does not adversely affect fetal, neonatal or childhood well-being and is safe to perform during pregnancy [2, 10, 14]. Since maternal exercise is beneficial for both mother and offspring, the millennia-old perspective of avoiding exercise during pregnancy has changed, and there has been a gradual positive shift in the scientific and clinical approach towards exercising and being physically active during pregnancy.

Reflecting gradual progress of the knowledge on maternal physical activity, American College of Obstetricians and Gynecologists has updated their initial committee opinion on physical activity in the years of 2002, 2015 and 2020 revising the content to incorporate evolving evidence on physical activity during pregnancy. The latest recommendations for women with uncomplicated pregnancies who have no contraindications to exercise have prescribed to accumulate 30–60 min of moderate intensity aerobic physical activity such as walking or dancing at least 3–4 days a week with a combination of resistance and stretch exercises [15]. These recommendations are more inclusive enabling pregnant women to involve in a wide range of activities that was vaguely suggested in the earliest version published in 1994 [16]. However, contact sports with high risk of abdominal trauma or imbalance, activities perform in supine position for a long period and scuba diving are still not recommended for women in pregnancy, especially after 20 weeks of gestation [15].

Despite the recommendations, most pregnant women in many countries are physically inactive [17–22], and the inactivity increases as the pregnancy progresses [23, 24]. Culture and cultural beliefs, misconceptions play a major role in the attitude towards exercise during pregnancy [24, 25]. Even today in some cultures, people still believe that the pregnancy is a time for rest and being sedentary [26]. The knowledge on maternal physical activity guidelines, safety concerns and attitudes of pregnant women towards physical activity during pregnancy are key determinants of exercise behavior in maternity [24, 27]. Lack of knowledge on physical activity during pregnancy act as a barrier for regular physical activity participation [25, 27]. Thus, the knowledge on maternal physical activity among pregnant women should be tested to identify the knowledge action gaps that would be advantageous in recognizing the current requirements in maternal education.

Accurate knowledge on physical activity during pregnancy would be beneficial of dispelling myths and misconceptions among pregnant women that encourage them to be active during pregnancy. Although previous studies were able to provide an insight in to the prevalence of knowledge on maternal physical activity, they were unable to provide regional differences and to identify the regions where evidence of knowledge on maternal physical activity is low or not yet empirically quantified. A majority of studies were solely based on individual countries and cross-country comparisons were lacking. Thus, a systematic review assessing the level of knowledge on maternal physical activity among pregnant women emphasizing potential regional differences has been a contemporary requirement in the field of obstetrics and gynecology. Up to date, there were no systematic review in the published literature fulfilled this requirement.

The primary aim of this proposed study is to systematically review literature that assesses the prevalence of knowledge on maternal physical activity among pregnant women in different regions.

## Methods

This systematic review is designed based on the guidelines for conducting systematic reviews of prevalence [28] and narrated following the recommendations in the latest statement of the preferred reporting items for systematic reviews and meta-analyses (PRISMA) [29].

### Search methods

Six scholarly databases including PubMed, Scopus, CINAHL Complete, SPORTDiscus with Full Text via EBSCO, Embase via OvidSP and Web of Science Core Collections will be searched electronically using keywords and index terms (MeSH) relevant to key concepts: physical activity, pregnant women and knowledge. Search terms such as exercise, yoga, motor activity, walking and sport will be used as synonyms for the main concept of physical activity. In addition, two antonyms for the concept of physical activity were included in order to make the search results more inclusive. Search query will include synonyms for pregnant women such as childbearing women, pregnancy, gestation, prenatal, antenatal and gravidity. Consciousness, awareness, comprehension, attitudes, beliefs, understanding, information and experience will be used as synonyms for knowledge. Most of these search terms had also been used in previous systematic reviews on physical activity in pregnancy [25, 27, 30]. Search query will be peer reviewed by a physical activity expert, public health specialist, research librarian and an obstetrician before applying in the databases. A comprehensive search will be conducted in each database applying search query that is specifically developed for individual database. An example of a search query developed for PubMed database is shown in Additional file 1. The time of publication will be restricted to the period from 2000 and beyond while no language restrictions will be applied. Articles will be filtered to remove the studies that have been conducted in species other than humans. If relevant studies are found in a language other than in English, service of a translator who is competent in both English and the language of the article will be sought to translate the content prior to data extraction.

Popular grey literature sources (e.g., Google Scholar, Mednar, CDC Stacks and Grey matters), digital open-source databases (e.g., OpenDOAR, OAIster and Nature Precedings), thesis collections (e.g., British Library Electronic Digital Thesis Online Service—EThOS, DART-Europe E-theses Portal, French Thesis-On-Line Repository, National ETD Portal, Global ETD Search, Indian doctoral theses database—Shodh, Thesis Canada Portal and Libraries Australia), relevant conference proceedings and research in progress (e.g., The Sigma Repository and Research Portfolio Online Reporting Tools, NIH—RePORT) will be searched to locate grey literature on the topic. Hand search of reference lists of the eligible articles will be conducted, and experts in maternal physical activity will be contacted to find more grey literature.

### Selection of studies

Citations of the literature search will be imported to the Zotero bibliography management software from the electronic databases and other sources. After removing the duplicates, two members of the review team will independently screen search results by reading the titles and abstracts of citations. Full texts of the studies that comply with inclusion criteria will be retrieved, and two reviewers will independently appraise content of the studies for eligibility. Studies those are not suitable for the review question will be excluded on the agreement of the two reviewers, and when a decision regarding the relevance of a study could not be attained between the two reviewers, the final decision will be made with consensus of a third reviewer.

### Inclusion criteria

Original cross-sectional, descriptive or observational quantitative studies published from year 2000 to 2022 will be included in the proposed systematic review. Studies that have quantified the prevalence of maternal physical activity knowledge or at least one of its components among pregnant women will be qualified for inclusion in this systematic review. Original studies from any country or region will be included in the review without any geographical restrictions. Studies carried out in special populations such as pregnant women with diseases or deprivation will also be included in the review but analyzed separately based on the severity of the condition.

### Exclusion criteria

Qualitative studies, studies carried out for the sole purpose of validating a tool, intervention studies, clinical trials, randomized control trials and case–control studies will be excluded. Studies that report physical activity knowledge among pregnant women as a combined outcome with the knowledge of other healthy lifestyle behaviors such as maternal nutrition or gestational weight gain will also be excluded. Articles published before the year of 2000 will be excluded as the knowledge, recommendations and guidelines on maternal physical activity had rapidly changed over time and studies published more than two decades ago are considered outdated and may not reflect the present situation.

### Quality assessment

The methodological rigor and risk of bias in each eligible study will be assessed by two independent reviewers using Joanna Briggs Institute Prevalence Critical Appraisal Tool, a critical appraisal tool designed to evaluate the methodological quality of studies reporting prevalence data [28, 31]. This tool provides a checklist to assess the sample size, representativeness of the sample, participant recruitment procedure, quality of reporting characteristics of study setting and participants, accuracy of data analysis, validity and reliability of measurements, and response rate [28, 31]. Any inconsistency of the judgment will be discussed among reviewers after re-evaluating the study quality by another independent member of the review team. However, no study will be excluded based on the methodological rigorousness, since even poor-quality studies might provide ample information on the topic at least to a certain extent [27]. The overall quality of the proposed review may not significantly affect by including poor-quality studies because the results of poor-quality studies will be reported separately and final conclusions of the review will only rely on medium to high quality studies.

### Data extraction

Selected studies for the systematic review will be read carefully by two reviewers to independently extract data on authors, year of publication, country of research, aims, study design, sample size, year of data collection, characteristics of participants, survey tools and key findings using a premade data collection sheet adapted from other similar systematic reviews [25, 30]. The data collection sheet will be pilot tested among reviewers to improve the reliability of data extraction process. Any incongruity or ambiguity of data extraction will be sorted by discussing with the other reviewer and/or contacting the original author of the article. The results and conclusions of each study will be scanned and extract information on the prevalence of maternal physical activity knowledge. These outcomes will be recorded laconically in the “key findings” section of the data extraction form.

### Outcomes

Knowledge on maternal physical activity is a broad concept and comprised of four main dimensions or levels [32]. Level 01: having simply heard that physical activity during pregnancy is good for maternal or fetal health and not harmful (e.g., safety concerns); Level 02: knowledge on specific health benefits of maternal physical activity (e.g., reduce the risk of cesarean deliveries, gestational diabetes mellitus, excessive gestational weight gain and maternal depression); Level 03: knowledge on maternal physical activity guidelines (e.g., frequency, intensity, type and time) and Level 04: knowledge about one’s own physical activity level during pregnancy and its associated risk to their own health. Thus, all above sub-categories of knowledge and any other dimension that is defined by the authors of the original studies as a component of knowledge on maternal physical activity will be considered as a primary outcome measure of this review. Different studies might have used different assessments to quantify the level of knowledge on maternal physical activity. However, the proposed review will only consider the prevalence of that measure in a defined pregnant population.

### Data analysis

Variables of overall knowledge and different subcomponents of knowledge will be separately analyzed. Similar (homogeneous) studies will be included in sub-groups or subsets considering the confounding factors such as geographical location. A narrative synthesis approach will be used to analyze the evidence in primary studies and summarize findings using text, words and manipulation of statistical data.

## Discussion

This review aims to systematically locate, assess and synthesis global evidence on the level of knowledge on maternal physical activity among pregnant women. Conclusions of the review will be made based on published and unpublished empirical evidence that coming from different regions in the world.

The rigorous and systematic methodology of this review is expected to provide firm evidence on the level of knowledge on maternal physical activity among pregnant women in different regions of the world and allows inter-regional comparisons. Findings will be profitable to identify research gaps and to locate regions that need further attention in terms of educating women on the benefits and recommendations on exercise and physical activity during pregnancy.

A certain degree of variation in the knowledge across regions are expected. If the variations are significant, it is also probable to identify reasons for regional differences which is advantageous in suggesting pragmatic strategies to improve knowledge on maternal physical activity in those identified regions. Further, the anticipated results of this review will be useful in tracing gaps in the knowledge and confusions about the benefits of maternal physical activity among pregnant women and provide potential solutions based on evidence. Findings of this proposed review will be useful in disseminating resources and funding opportunities prioritizing the regions that need immediate attention of improving knowledge on maternal exercise.

Strengths of this review are that this will be conducted based on previously published guidelines [28] and recommendations in the PRISMA statement [29]. Major academic databases related to the topic will be systematically searched for relevant articles while missing studies on the topic and grey literature will be located meticulously. As recommended in the series on systematic review from Joanna Briggs Institute, two authors will involve independently in the screening of search results, study selection, critical appraisal of studies and in the process of data extraction [33, 34]. The use of felicitous tools and guidelines in data collection and reporting would generate trustworthy conclusions and recommendations on the topic that is beneficent to policymakers, health educators and clinicians. Although this review will be conducted systematically, it is not without limitations that should be cautious when interpreting the results. The main limitation of this review is that some pertinent evidence in the grey literature may be difficult to locate based on the nature of the library platform, level of public availability and time restrictions. Despite the limitations, this is the first review that provides a world-wide overview of the prevalence of knowledge on maternal physical activity among pregnant women.

## Supplementary Information

**Additional file 1**: Search strategy—an example of a search strategy developed for PubMed database.

## Data Availability

Data sharing is not applicable to this article as no datasets were generated or analysed during the current study.
