# Supplementary material for: Prevalence of knowledge on maternal physical activity among pregnant women: a protocol for a systematic review
Source: J Act Sedentary Sleep Behav. 2022 Nov 2;1:7. doi: 10.1186/s44167-022-00006-0 (PMC11934496; doi:10.1186/s44167-022-00006-0)
Supplement: Supplementary file 1 — Additional file 1: Search strategy—an example of a search strategy developed for PubMed database. [file 44167_2022_6_MOESM1_ESM.docx]

**Additional file 1**

Concept 1: Physical Activity

Keywords: physical activity(ies), exercise, yoga, motor activity(ies), walk(ing), inactivity, sport, sedentary behavior

Concept 2: Pregnant Women

Keywords: pregnant women, childbearing women, pregnancy(ies), pregnant, gestation(al), prenatal, antenatal, gravidity

Concept 3: Knowledge

Keywords: knowledge, consciousness, awareness, comprehension, attitudes, beliefs, understanding, information, experience

(("physical activit*"[Title/Abstract] OR "exercise"[Title/Abstract] OR "yoga"[Title/Abstract] OR "motor activit*"[Title/Abstract] OR "walk"[Title/Abstract] OR "inactivity"[Title/Abstract] OR "sport"[Title/Abstract] OR "sedentary"[Title/Abstract]) AND ("pregnant women"[Title/Abstract] OR "childbearing women"[Title/Abstract] OR "pregnancy"[Title/Abstract] OR "pregnant"[Title/Abstract] OR "gestation"[Title/Abstract] OR "prenatal"[Title/Abstract] OR "antenatal"[Title/Abstract] OR "gravidity"[Title/Abstract]) AND ("knowledge"[Title/Abstract] OR "consciousness"[Title/Abstract] OR "awareness"[Title/Abstract] OR "comprehension"[Title/Abstract] OR "attitudes"[Title/Abstract] OR "beliefs"[Title/Abstract] OR "understanding"[Title/Abstract] OR "information"[Title/Abstract] OR "experience"[Title/Abstract])) AND ((humans[Filter]) AND (2000/1/1:2022/12/31[pdat]))
